# Bio‐Inspired Internal Stress Engineering for Microscale Surface Cracking in Sensitive Strain Sensors

**DOI:** 10.1002/advs.77961

**Published:** 2026-09-27

**Authors:** Jae Yeong Jang, Hanchul Cho, Young Jung

**Affiliations:** ^1^ Major of Mechanical Engineering Pukyong National University Busan Republic of Korea; ^2^ Department of Intelligent Robot Engineering Pukyong National University Busan Republic of Korea; ^3^ Precision Mechanical Process and Control R&D Group Korea Institute of Industrial Technology Busan Republic of Korea

**Keywords:** bio‐inspired internal stress, high sensitivity, microscale cracks, strain sensor, thermally expandable microspheres

## Abstract

The fabrication of microscale cracks on flexible substrates is a crucial step for potential applications such as human–machine interfaces, biosignal analysis, and structural health monitoring. However, existing crack fabrication methods predominantly rely on externally applied mechanical stimuli such as stretching or bending, which limits the freedom with which cracks can form in terms of their location, density, and geometry. In this study, we introduce a bio‐inspired internal stress engineering strategy that enables tunable microscale cracks to be driven by internal stress induced through microsphere expansion. Microscale cracks can be reproducibly formed by exploiting the localized stress concentration and thickness‐dependent mechanical property transition of an ultrathin metal film. The crack‐based strain sensor exhibits an ultrahigh sensitivity of 24 320 at a strain of 1.6%. The proposed strategy demonstrates excellent sensing performance under both static and dynamic loading conditions, including high repeatability and frequency stability, demonstrating its considerable potential for practical sensing applications. Further, we demonstrate its applicability in wearable sensors for real‐time physiological monitoring and structural health monitoring of aircraft structures to verify its versatility across diverse industrial fields.

## Graphical abstract

This study presents a bio‐inspired internal stress engineering strategy to fabricate highly sensitive crack‐based strain sensors. By harnessing the internal expansion energy of microspheres and the thickness‐dependent brittle‐to‐ductile transition of ultrathin gold films, tunable microscale cracks are generated. The resulting sensors exhibit an ultrahigh gauge factor, enabling real‐time wearable physiological monitoring and structural health monitoring for aerospace applications.

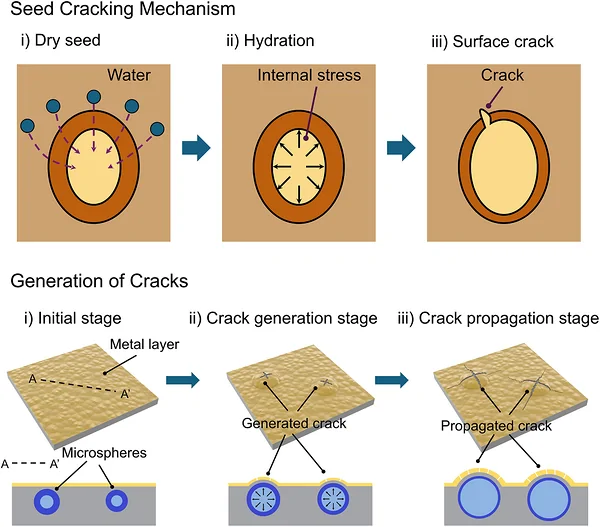

## 1 Introduction

Flexible strain sensors have recently attracted significant attention for analyzing mechanical deformation in real‐time human‐motion interfaces, biosignal analysis, and structural health monitoring [1, 2, 3, 4, 5]. Achieving the full potential of these applications requires a high gauge factor (*GF*) in the low‐strain range. For these novel applications, the *GF* is a key performance indicator for subtle strain sensing, representing the ratio of the relative change in electrical resistance to applied mechanical strain. The required high *GF* can be achieved by utilizing advanced sensing materials with superior intrinsic electromechanical properties [6] or engineering structural designs that amplify responses under minimal strain. The former can be exemplified using graphene‐based nanocomposites possessing extraordinary intrinsic electron mobility and high aspect ratio. However, these materials face challenges such as poor interfacial bonding or nonuniform dispersion. These issues can limit their practical application in large‐scale device fabrication. Conversely, the latter strategy focuses on structural geometry instead of being restricted to specific material sets. Researchers can achieve superior performance by employing structural geometries such as gap openings [7, 8], percolation networks [9], or biomimetic cracks [8, 10, 11, 12, 13, 14, 15]. This approach has attracted significant interest and enabled diverse applications in recent years.

Microcracks have emerged as a promising approach among various structural strategies because stress concentration and crack opening–closing behavior in conductive thin films can induce large resistance changes even under subtle mechanical deformation [11, 13, 14, 15]. Conventional approaches generally employ external stimuli, such as mechanical bending or stretching of the substrate, to generate surface cracks in conductive films. Under appropriate external stimuli conditions, these methods can produce sensing‐active crack networks with a preferred orientation, resulting in reproducible resistance changes during strain sensing. Among various conductive metals, Au is particularly attractive for sensing applications because of its high electrical conductivity. However, generating a sensing‐active crack network in supported Au films through conventional external stimuli can be challenging under certain film and substrate conditions [11, 14]. Moreover, conventional crack‐generation methods generally require a pre‐deformation process and provide limited control over crack density and morphology. Therefore, an alternative strategy capable of generating microscale cracks in Au films without macroscopic pre‐stretching or bending is desirable. This approach provides unprecedented degrees of freedom in the fabrication process, which helps realize highly sensitive Au‐based strain sensors that were previously unattainable.

In this study, we introduce a bioinspired internal stress engineering strategy for fabricating tunable microscale cracks driven by internal stimuli, termed microscale cracks induced through microsphere expansion (MCME). This approach was inspired by the seed germination process, wherein the internal swelling pressure induces sufficient stress to break through a rigid outer shell. We utilized the localized stress concentration from the expanding microspheres and the thickness‐dependent brittle‐to‐ductile transition of Au films by mimicking this phenomenon. Using this approach, we successfully induced sharp crack initiation even in ultrathin Au films. This mechanism enabled highly sensitive strain sensing in the subtle‐strain range, which helped leverage the opening and closing of microscale cracks. The MCME‐based strain sensors exhibit a high *GF* of up to 24 320 at a strain of 1.6%. Their sensing performance was validated under static and dynamic loading conditions, demonstrating excellent stability, repeatability, and reliability. Based on their highly sensitive signal output, the proposed sensors were applied successfully to biosignal monitoring devices, including voice recognition, swallowing, and pulse detection under both normal and post‐exercise conditions. Furthermore, their versatility was demonstrated through reliable structural health monitoring using a strain sensor array integrated into notched carbon fiber‐reinforced plastic (CFRP) for aircraft structures.

## 2 Results and Discussion

### 2.1 Concept and Mechanism of Microscale Cracks Induced Through the Microsphere Expansion (MCME) Strategy

**FIGURE 1:**
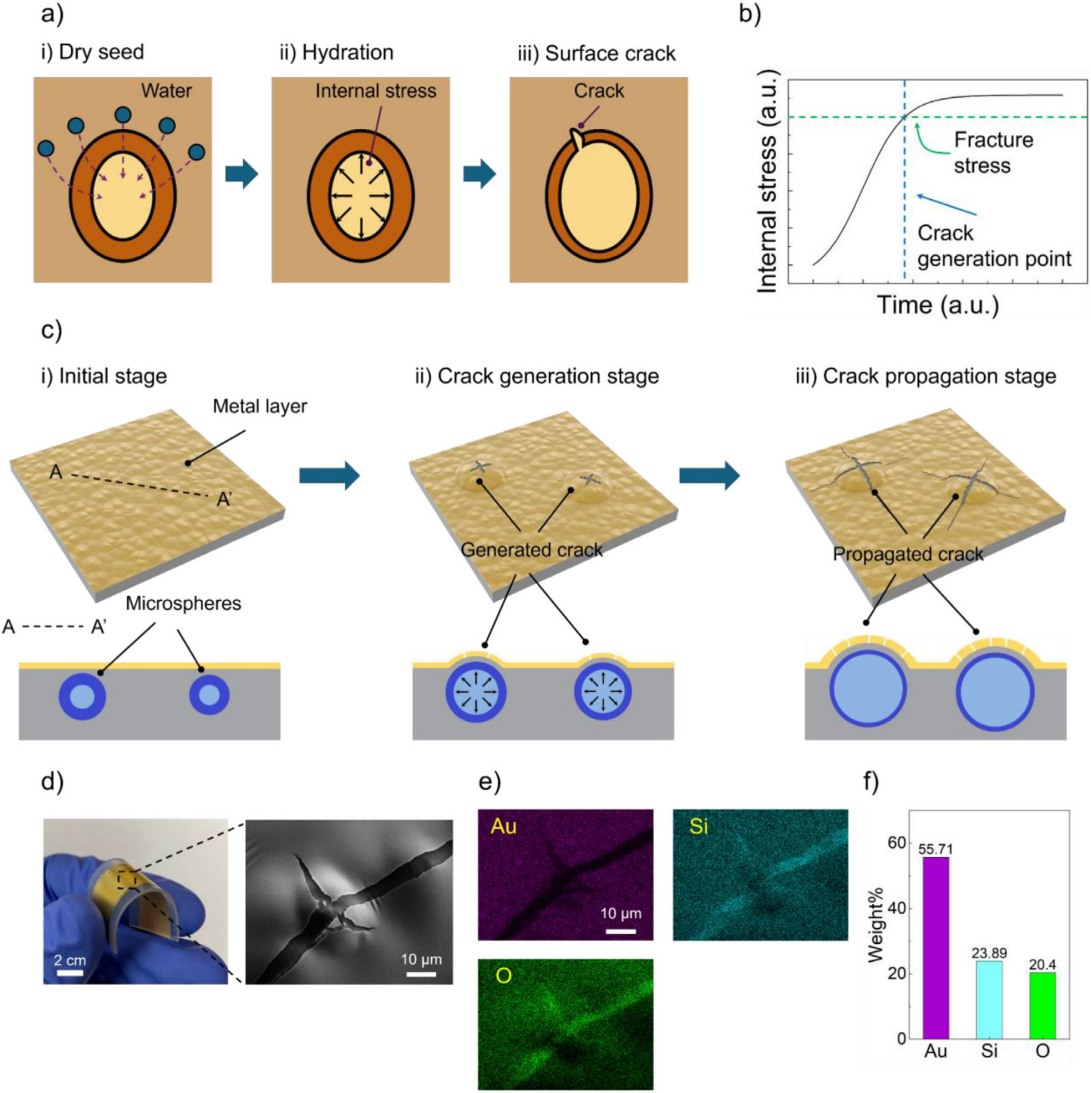
Bio‐inspired internal stress engineering from tunable microscale crack fabrication. (a) Schematic of seed cracking mechanism inspired by hydration. Water absorption leads to internal stress accumulation, eventually causing surface cracking. (b) Internal stress vs. degree of microsphere expansion. Crack generation occurs when the internal stress exceeds the fracture threshold. (c) Schematic displaying the generation of a microscale crack via internal stress by microsphere expansion (MCME): (i) Initial state showing a flat surface with no internal stress; (ii) crack generation triggered by microsphere expansion; (iii) crack propagation driven by localized stress concentration. (d) Photograph of the fabricated MCME film. (e) Scanning electron microscope (SEM) image of the microscale cracks. (f) Energy‐dispersive x‐ray spectroscopy (EDS) mapping results of the cracked region indicating the elemental composition (Au, Si, and O) at the cracked region, which confirms silicone‐based material composition.

Figure 1 illustrates bio‐inspired internal stress engineering for fabricating tunable microscale cracks using internal stimuli. This mechanism is inspired by the natural germination of seeds during hydration, where water absorption generates an internal stress that causes surface fractures (Figure 1a). The seed includes a rigid outer shell and an internal core. Upon hydration, the internal core absorbs water and undergoes volumetric expansion (Figure 1a i). The volumetric expansion of the internal shell leads to internal stress accumulation at the rigid outer shell (Figure 1a ii). As depicted by the stress–time relationship in Figure 1b, the accumulated internal stress eventually reached a critical threshold known as fracture stress. At this moment, a crack formed on the surface of the shell at its weakest point, which enabled the embryo to emerge and grow (Figure 1a iii). We applied this mechanism to fabricate an MCME strategy for inspiring this natural phenomenon in an engineering process. Figure 1b illustrates the evolution of the internal stress as a function of the degree of microsphere expansion. As the internal stress increases gradually, it eventually reaches the fracture stress of the metal film. Surface cracks are initiated at this critical point, which is defined as the crack initiation point. Figure 1c exhibits the detailed crack generation mechanism. As shown in the initial state (Figure 1c i), a thin metal layer (Au) is deposited onto a soft substrate mixed with expandable microspheres. Expandable microspheres include thermoplastic resins (acrylonitrile copolymers) in an outer shell and liquid hydrocarbon (isopentane) in the inner core (Figure S1). When an expandable microsphere is subjected to a certain temperature, it expands by three to six times its initial size [16]. During the crack‐generation stage (Figure 1c ii), an external stimulus causes the microspheres to expand, mimicking the internal swelling of the seed. This expansion creates a localized stress concentration at the apex of each microsphere, which directly strains the overlying Au thin film. In the crack propagation stage (Figure 1c iii), initiated cracks propagate radially when the microspheres reach their maximum expansion, forming an interconnected network of microscale surface cracks. When cooled to room temperature, the thermoplastic shell solidifies again and retains its expanded microsphere morphology. This preserves the dome‐shaped deformation and crack structure induced by the microspheres in the overlying Au film. The retention of the expanded morphology was further confirmed by scanning electron microscope (SEM) images obtained after 10 days, in which the microsphere‐induced surface 3D structures remained clearly visible without apparent collapse (Figure S2).

The physical and chemical characteristics of the resulting MCME films are verified as shown in Figure 1d–f. Figure 1d shows a photograph of the fabricated MCME film, highlighting its flexibility in fabrication based on soft elastomers. The SEM image reveals the detailed morphology of cracks, which are sharp and well‐defined and originate from the peak of the underlying microsphere (Figure 1e). Energy‐dispersive x‐ray spectroscopy (EDS) mapping (Figure 1f) is used to confirm the elemental compositions of the microcracks. The EDS mapping results exhibit crack formation: while Au (55.71 wt.%) remains the dominant element across the overall surface, Si (23.89 wt.%) and O (20.4 wt.%) are clearly observed at the specific points of microsphere expansion. This localized detection of Si and O within the crack regions confirms that internal stimuli from the microspheres successfully fracture the overlying Au layer, exposing the underlying substrate surface. These morphological and elemental analyses confirm the successful realization of the bioinspired MCME strategy.

### 2.2 Theoretical and Experimental Investigation of the Crack Generation Mechanism

**FIGURE 2:**
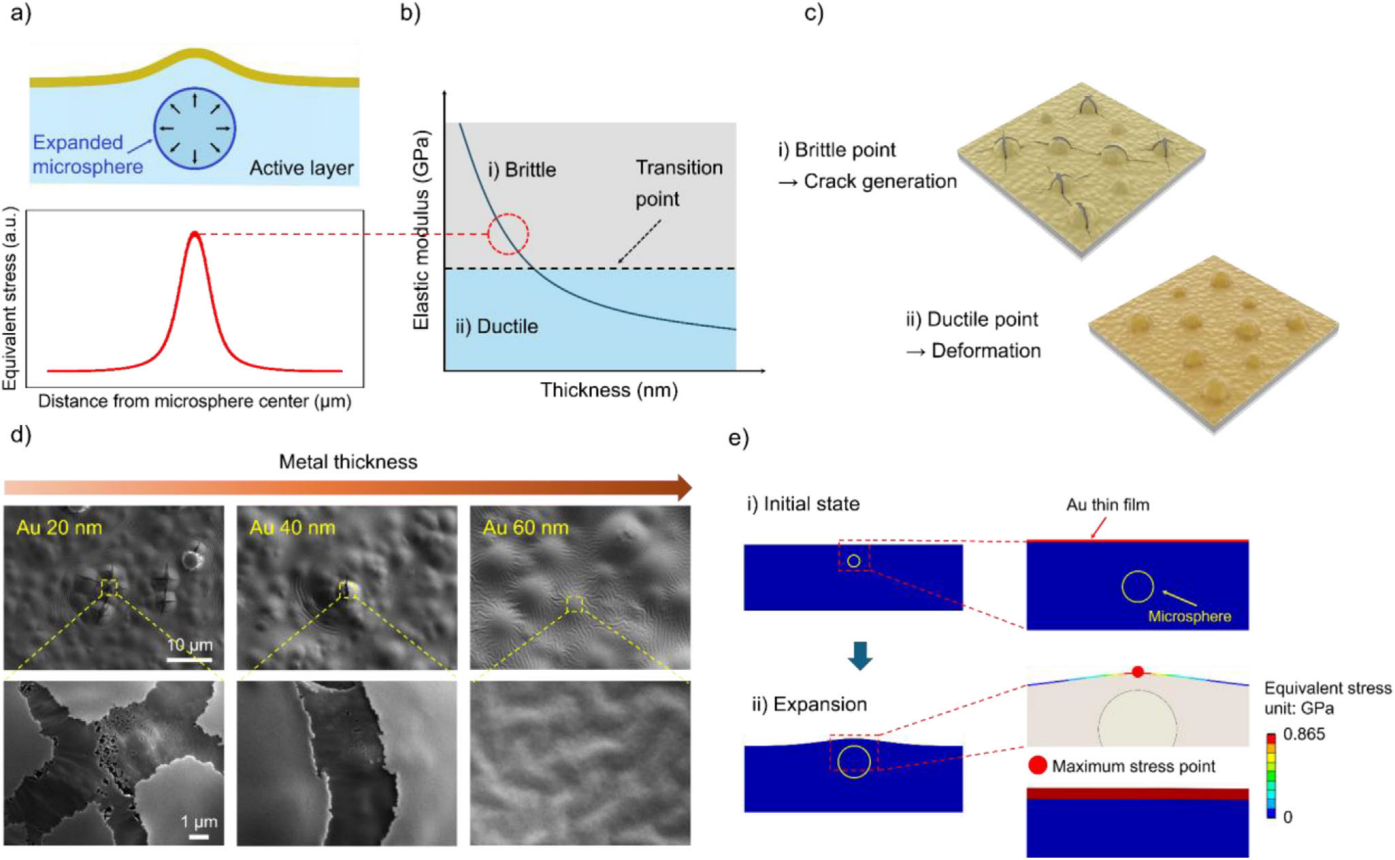
Thickness‐dependent mechanical property transition and crack initiation. (a) Schematic illustration of the localized stress distribution induced by microsphere expansion. (b) Schematic of the thickness‐dependent transition of Au thin films from brittle to ductile behavior. Crack initiation occurs when the film thickness enters the brittle regime near the transition point. (c) Schematic of surface responses in different mechanical regimes: (i) in the ductile regime, microsphere expansion results in surface deformation without fracture; (ii) in the brittle regime, localized stress concentration leads to surface crack generation. (d) SEM images of Au thin films with varying thicknesses (20, 40, and 60 nm), demonstrating a transition from distinct crack formation to wrinkling patterns. (e) Finite element analysis of the microsphere‐induced localized stress in the Au thin film, which confirms that the maximum equivalent stress is concentrated directly above the microsphere.

Figure 2 depicts the fundamental mechanism of surface crack generation driven by internal microsphere expansion and thickness‐dependent mechanical properties of Au thin films. Traditionally, crack‐based strain sensors have relied on brittle materials such as Pt because their surfaces readily fracture under external mechanical stimuli such as bending or stretching. Although Au is a superior candidate for flexible electronics because of its exceptional electrical conductivity and chemical stability, its inherent ductility inhibits the formation of well‐defined cracks necessary for high‐sensitivity sensing.

In this study, we overcome this limitation by strategically utilizing the microsphere‐induced localized internal stress coupled with the thickness‐dependent brittle‐to‐ductile transition of Au thin films. As illustrated in Figure 2a, microsphere expansion generates a localized stress distribution in the overlying metal film. The equivalent stress is greatest near the center of the microsphere‐induced deformation and decreases gradually with increasing radial distance. The mechanical response of the Au film to this localized stress is strongly governed by its thickness. As indicated in Figure 2b, Au exhibits a distinct transition point in its mechanical behavior. Above a specific threshold thickness, Au maintains its bulk‐like ductile characteristics [17, 18]. In this regime, it exhibits a relatively lower elastic modulus, favoring elastic deformation over fracture. Below this threshold, Au transitions into a brittle state with a significantly higher elastic modulus in the ultrathin regime. This transition is crucial because it determines if internal stimuli result in simple surface deformation or the initiation of sensing active cracks (Figure 2c).

The experimental validation of this transition is provided by the SEM images in Figure 2d. The 20‐nm‐thick Au film resides within the brittle regime, which results in the formation of microscale cracks driven by the internal stress induced by microsphere expansion. As the thickness increases to 40 nm, cracks are generated in the Au film; however, the crack density decreases significantly. This result indicates a shift toward the ductile regime. In addition, at a thickness of 60 nm, the deformation behavior changes completely. Instead of fracturing, the film exhibits wrinkling patterns. This thickness‐dependent morphological transition is further supported by the electrical measurements (Table S1). After microsphere expansion, the resistance of the 20 nm Au film became unmeasurable. In contrast, the resistance of the 40 nm film increased by approximately 10.4 times (from 1.98 to 20.5 Ω), while the resistance of the 60 nm film changed only slightly (from 2.08 to 2.93 Ω). These results indicate that the microsphere‐induced localized internal stress progressively shifts from crack‐dominated fracture toward wrinkle‐dominated deformation as the Au film thickness increases. To quantitatively evaluate these morphological changes, the crack density was compared as a function of microsphere concentration, metal‐film thickness, and metal type (Figure S3). The results demonstrate that microscale crack formation is strongly governed not only by the microsphere concentration but also by the type and thickness of the metal film. In contrast, distinct crack networks were not observed in the 40‐ and 60‐nm‐thick Pt films under the same microsphere‐expansion conditions. As shown in Figure 2e, we conduct a finite element analysis (FEA) to investigate localized stress distribution. The simulation model includes a soft elastomer, an embedded microsphere in its initial state, and an Au thin film on the top surface (Figure 2e i). The results confirm a relatively high equivalent stress concentration at the interface upon the threefold expansion of the microspheres (Figure 2e ii). The maximum stress was observed at the apex of the microsphere directly beneath the Au thin film. Additional FEA showed that increasing the microsphere depth from 15 to 35 µm reduced the maximum stress transferred to the Au film, indicating that microsphere position within the active layer also influences crack initiation (Figure S4). The calculated equivalent stress in this region exceeded the fracture threshold of the thin Au film, providing a theoretical explanation for the site‐specific localization of crack initiation at the center point of the microsphere. This synergy between the engineered brittleness and localized internal stimuli enables the fabrication of highly sensitive crack‐based sensors that were difficult to achieve with Au.

### 2.3 Fabrication of MCME‐Based Strain Sensors

**FIGURE 3:**
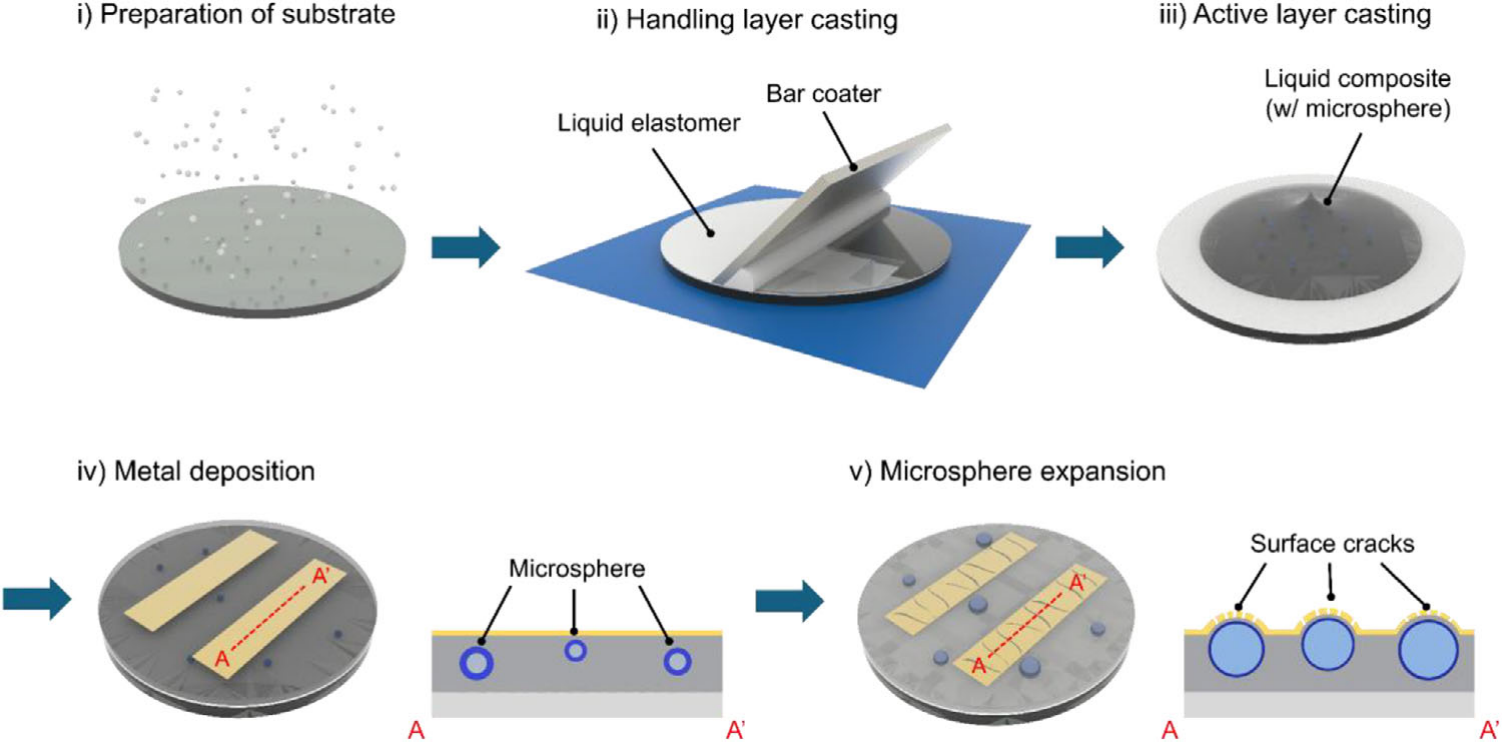
Fabrication procedure of MCME‐based strain sensors. (i) Self‐assembled monolayer (SAM) coating on the substrate to prevent elastomer adhesion. (ii) Casting of the liquid elastomer onto the substrate for handling. (iii) Spin coating of composite mixtures uniformly onto the handling layer. (iv) A thin metal film is deposited onto the composite surface. (v) Microsphere expansion is induced by thermal treatment, which results in tunable microscale cracks of the metal layer caused by internal stress.

Figure 3 illustrates the detailed fabrication process of MCME‐based strain sensors. A self‐assembled monolayer (SAM) was initially coated on the base substrate to prevent adhesion during the subsequent processing steps (Figure 3 i). A 0.2‐mm‐thick handling layer composed of a soft elastomer (Dragon Skin 30) was cast onto the treated substrate using a bar coater to ensure a uniform and mechanically compliant substrate (Figure 3 ii). Subsequently, an active layer consisting of a liquid composite mixture of polydimethylsiloxane (PDMS) and thermally expandable microspheres (TEMS) was cast onto the handling layer. This composite was spin coated to achieve a uniform thickness of ∼ 100 µm (Figure 3 iii). After the composite was fully cured, a thin Au film was deposited onto its surface in a predefined pattern using a shadow mask (Figure 3 iv). As illustrated in the cross‐sectional schematic (A–A′), the metal film exhibits high uniformity prior to the application of internal stimuli. Finally, the metal‐deposited composite film is subjected to thermal treatment at 170°C for 5 min, which causes the microspheres inside the soft elastomer to expand (Figure 3 v). This volumetric expansion generates substantial internal stress within the composite layer. A localized stress concentration is induced in the overlying metal film as the microspheres expand within the soft elastomer, thereby leading to the formation of microscale cracks. Figure S5 shows the generation and propagation of microscale cracks with microsphere expansion. SEM analysis further showed that the area fraction of microsphere‐associated surface features increased with increasing microsphere concentration (Figure S6), whereas the expanded microspheres exhibited comparable size distributions at 1, 3, and 5 wt.% (Figure S7). These results indicate that the concentration‐dependent morphological changes mainly arise from an increased microsphere population rather than a change in the characteristic microsphere size. This fabrication process enables the formation of tunable microscale crack networks by controlling the microsphere concentration and metal‐film thickness.

### 2.4 Sensing Performance of MCME‐Based Sensitive Strain Sensor

**FIGURE 4:**
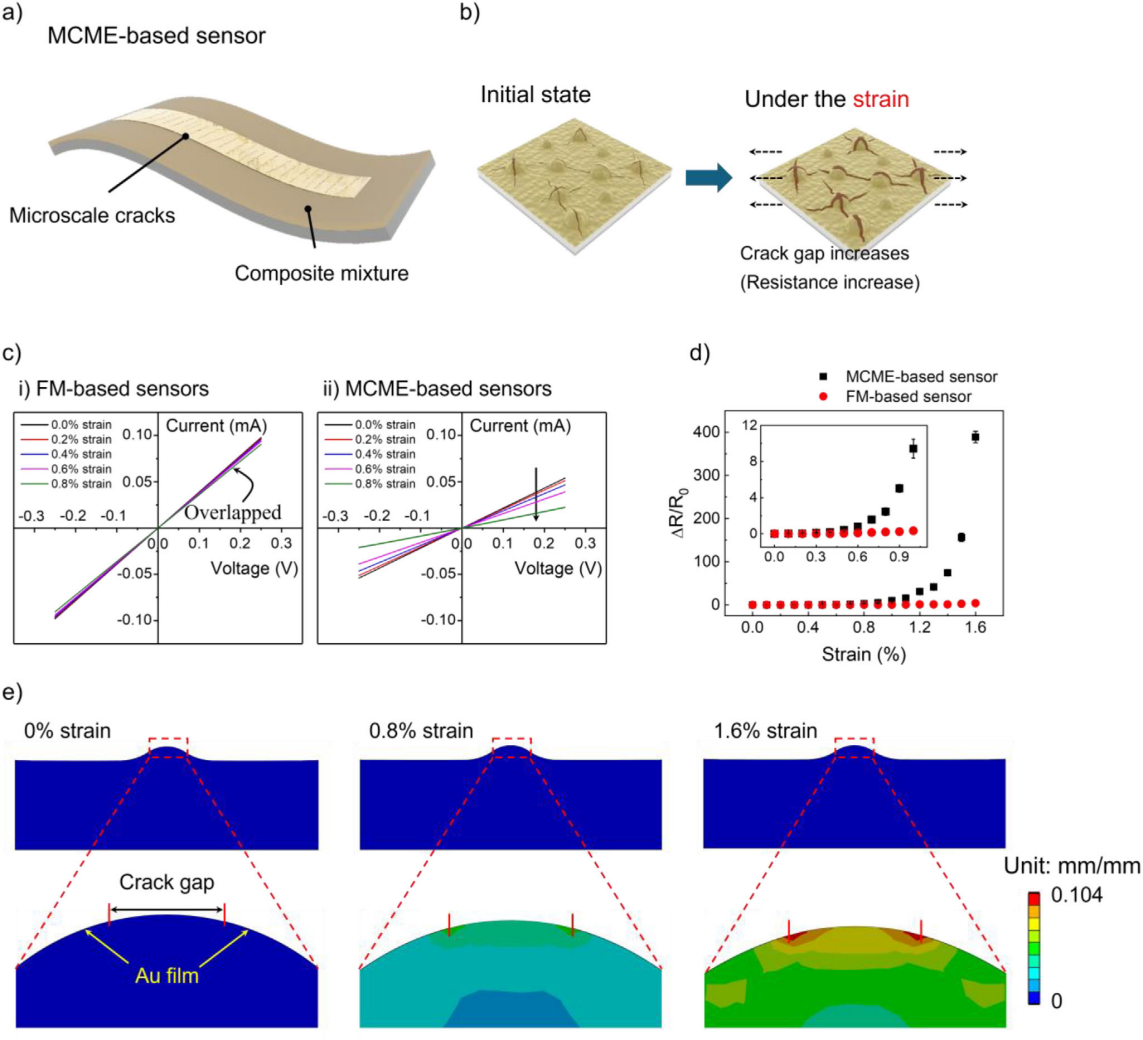
Working principle and sensing performance of MCME‐based strain sensors. (a) The structure of MCME‐based strain sensors. (b) Working principle of a highly sensitive strain sensor. When strain is applied, the crack gap opens, which results in a rapid change in the electrical resistance of the metal film. (c) Current–voltage (I–V) characteristics of the strain sensors with and without microscale cracks: (i) Flat metal (FM)‐based strain sensor shows negligible resistance change under strain; (ii) MCME‐based sensor exhibits a significant resistance variation with increasing strain. (d) Resistance change (Δ*R*/*R*_0_) vs. applied strain for microscale crack (black squares) and flat metal (red circles) sensors. (e) FEA simulation results show the local strain distribution.

Figure 4a presents a schematic of the highly sensitive, flexible, and soft strain sensor, in which a metal film featuring microscale cracks serves as the active sensing layer. The proposed MCME‐based strain sensor is composed of a composite mixture and a conductive metal film. This simple material composition facilitates a cost‐effective and facile fabrication process. The working principle of the strain sensor is illustrated in Figure 4b. A localized stress concentration occurs at the tips of microscale cracks when an external strain is applied to the sensor. Consequently, the cracked thin film undergoes a significantly larger deformation than the uniform thin film. This deformation triggers the opening of cracks, effectively narrowing the conductive pathways for electron transport. This mechanism leads to a rapid change in electrical resistance, even under subtle deformations, achieving high sensitivity under an applied strain.

As shown in Figure 4c, we conduct current–voltage (I–V) measurements to intuitively evaluate the effect of microscale cracks on sensing performance. The measurements were performed by sweeping the voltage from −0.25 to 0.25 V. According to Ohm's law, the slope of the I–V curve represents the inverse of the resistance; thus, a decrease in slope indicates an increase in resistance. For a flat metal (FM)‐based sensor (indicating a uniform thin film), there is no significant change in the slope when the strain is increased from 0% to 0.8%, resulting in nearly overlapping I–V curves. In contrast, the MCME‐based strain sensor exhibits a distinct and rapid decrease in the slope with an increase in strain. These results demonstrate that the MCME‐based sensor is highly sensitive and produces a significant change in resistance in response to mechanical deformation. We measure the sensitivity of the strain sensors under different strain (Figure 4d). The *GF* can be calculated by *GF = δ*(Δ*R*/*R*_0_)/*ε*, where Δ*R*, *R*_0_, and *ε* represent the resistance change, initial resistance, and applied strain, respectively. The black squares and red circles represent the measurement results of the MCME and FM‐based sensors, respectively. The change in the resistance of the sensor was measured at a maximum strain of 1.6%. The FM‐based sensor exhibited a negligible resistance change of 0.33 and low *GF* (33), even at 1.0% strain. In contrast, for the MCME‐based sensor, we observed that the change in resistance was initiated at a strain of ∼ 0.5% and increased rapidly. The response curve exhibited exponential growth, with the slope increasing sharply with an increase in strain. The sensor sensitivity increased by ∼ 12.38, 30.24, and 96.44 times at applied strains of 0.8%, 1.2%, and 1.6%, respectively. For further comparison, a mechanically cracked strain sensor was fabricated with the same gold thickness, substrate, and device geometry, and was subjected to 60% pre‐strain. However, the resulting excessively dense and irregular crack network severely disrupted the conductive pathways, rendering the sensor unreliable (Figure S8).

The FEA simulations were conducted to investigate the local strain distribution and the relationship between the deformation behavior and resulting high sensitivity (Figure 4e). Cracked geometries were modeled based on actual SEM images. Tensile deformation was applied symmetrically to both lateral sides of the model, while the bottom surface was constrained in the vertical direction. The normal elastic strain distribution was analyzed to understand the strain distribution of microscale cracks. Under tensile strain, most of the strain was concentrated in the cracks formed at the point of maximum microsphere expansion. This resulted in a relatively local strain distribution with a maximum local strain of 10.4% occurring at the cracked surface under an applied strain of 1.6%. This localized strain distribution indicates that the conductive pathway changed effectively, leading to a higher sensitivity according to strain than that of a uniform thin film.

**FIGURE 5:**
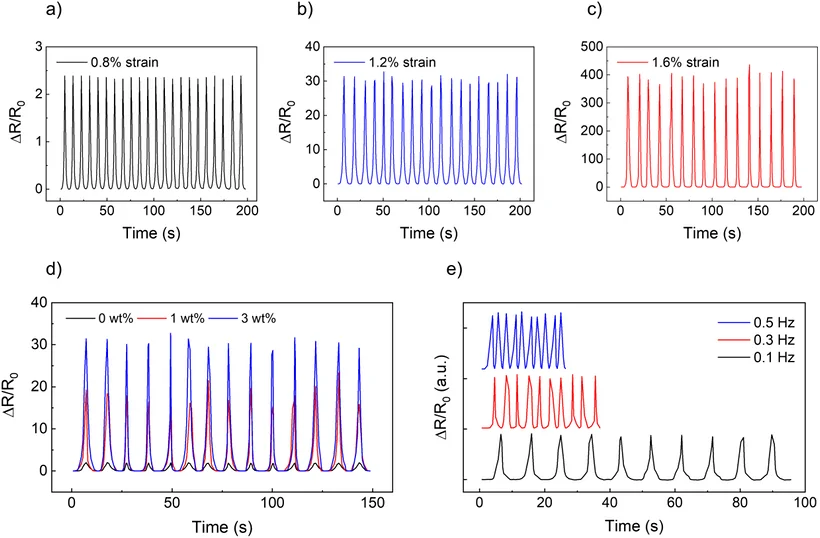
Sensing performance of MCME‐based strain sensors. (a–c) Dynamic response of the MCME‐based sensor under various applied strains at (a) 0.8%, (b) 1.2%, and (c) 1.6% strain. A *GF* of ∼ 24 320 is achieved at 1.6% strain, demonstrating ultrahigh sensitivity. (d) Comparison of strain sensitivity for sensors with varying microsphere concentrations (0, 1, and 3 wt.%). (e) Frequency response of the sensor under 0–0.8% strain at 0.1, 0.3, and 0.5 Hz. The MCME‐based sensor exhibits consistent and reliable performance across different strain rates.

The sensing performance of the proposed strain sensor is evaluated under static and dynamic strain loadings (Figure 5). Figure 5a–c present changes in the resistance of the sensors during cyclic loading–unloading at different applied strains (0.8, 1.2, and 1.6%). The measured normalized resistance variation (Δ*R*/*R*_0_) exhibited a sharp increase with increasing strain. The *GF*s were calculated to be 1275 and 2617 at 0.8% and 1.2% strains, respectively. A high *GF* of ∼ 24 320 was achieved at a strain of 1.6%. In case of 2.0% strain, the gaps in the microscale cracks increased, resulting in unreliable and inconsistent resistance variation (Figure S9). In addition, we compared the dynamic response of the FM and MCME‐based sensors. The MCME‐based sensor exhibits a significant resistance variation of 306, which is 12 times higher than that of the FM‐based sensor (Figure S10). These results confirmed that MCME‐based sensors possessed an exceptional capability to accurately detect subtle strains. In addition, these ten different MCME‐based sensors exhibited similar changes in the responses, with negligible deviations (Figure S11). Comparable expanded microsphere size distributions were observed both within a single sample and among independently fabricated samples, with mean diameters of approximately 21–22 µm (Figure S12). This result further supports the reproducibility of the fabrication process. We further investigated the effect of the microsphere concentration (0, 1, and 3 wt.%) on sensor sensitivity at a fixed strain of 1.2% to optimize sensing performance (Figure 5d). The sensor fabricated with 3 wt.% microspheres exhibited a significant resistance change of 30 and maintained stable signal reproducibility under repeated strain cycles. In contrast, sensors with lower microsphere concentrations (0 and 1 wt.%) exhibited negligible or relatively low resistance changes because of insufficient crack density. The MCME‐based strain sensor with a higher microsphere concentration of 5 wt.% exhibited increased sensitivity (Figure S13). However, its reliable sensing range was reduced to approximately 1.2% strain due to excessive crack formation and disruption to the conductive pathways. In the case of Pt, the Pt‐based sensor exhibited a progressive increase in resistance variation during repeated loading, indicating unstable evolution of the conductive network and poor signal repeatability (Figure S14). Consequently, the combination of a 3 wt.% microsphere concentration and an Au film was selected as the optimal condition for further evaluation and functional applications. Further, we investigated the dynamic sensing performance of the MCME‐based strain sensor to verify its stability. Figure 5e displays measurement results under different frequencies of 0.1, 0.3, and 0.5 Hz at a strain of 0.8%. The sensor exhibited excellent stability and consistent signal patterns regardless of frequency changes. The 10% to 90% rising time (*t_r_*) and 90% to 10% falling time (*t_d_*) were measured as 305 and 416 ms, respectively (Figure S15). We also calculated the limit of detection (LOD) of our sensor, as shown in Figure S16. The theoretical LOD of our sensor was calculated as low as 0.070% strain. Long‐term cyclic stability was further evaluated over 5000 loading–unloading cycles at 0.7% strain (Figure S17). The average peak Δ*R*/*R*_0_ changed from 1.563 ± 0.026 during the initial ten cycles to 1.653 ± 0.082 during the final ten cycles, corresponding to a modest drift of approximately 5.76%, with no abrupt signal loss or electrical failure. Finally, to evaluate the overall sensing performance relative to previously reported works, the MCME‐based strain sensor was compared with recently reported micro/nanoscale crack‐based strain sensors in Table S2 [8, 19, 20, 21, 22, 23, 24, 25, 26]. The comparison demonstrates that the proposed sensor offers a favorable combination of high sensitivity, sensing range, reliability, and fabrication simplicity.

### 2.5 Application of Biosignal Monitoring Systems

**FIGURE 6:**
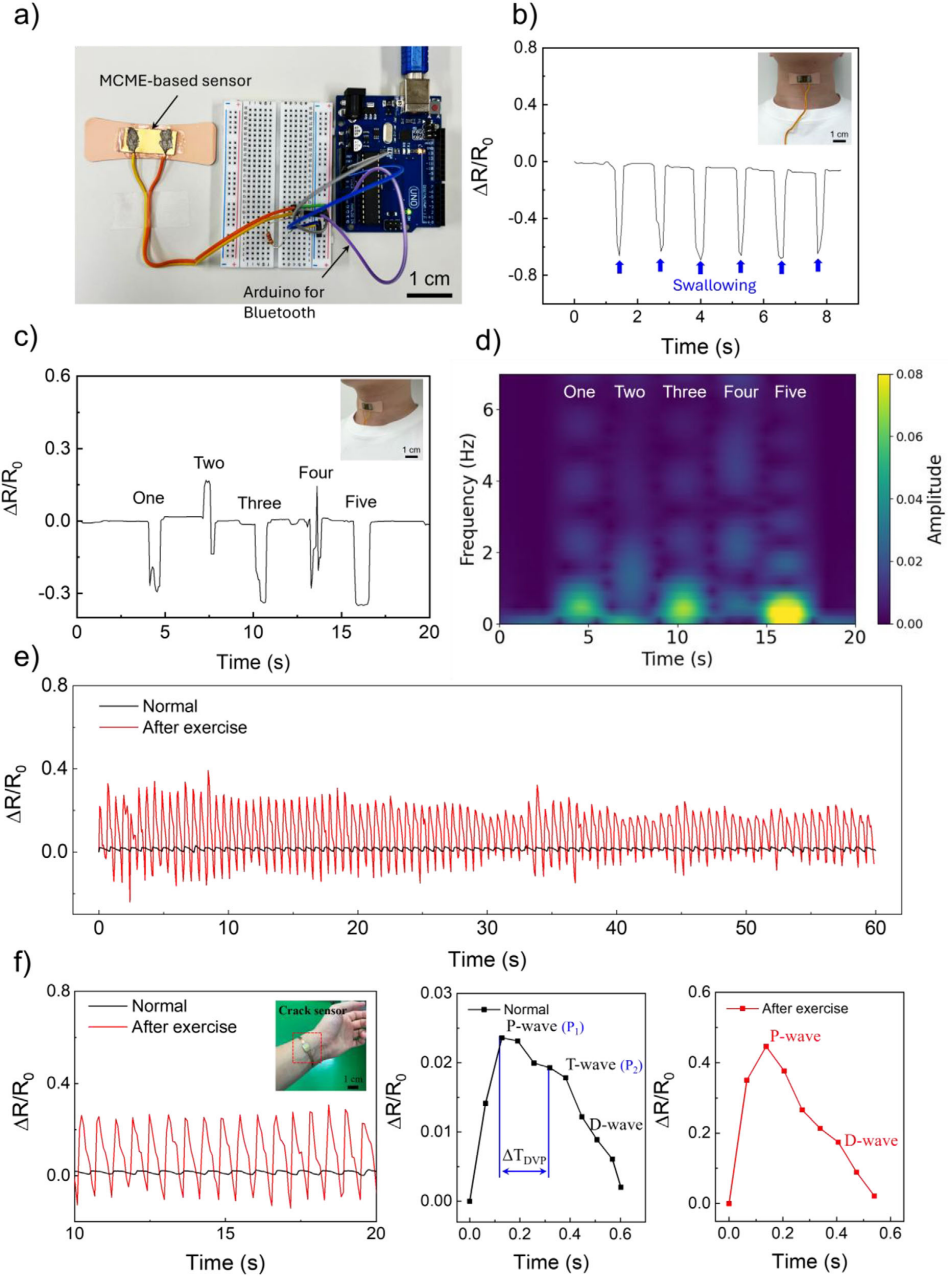
Application of the MCME‐based strain sensor for wearable physiological monitoring. (a) Photograph of the fabricated wearable device integrated with an Arduino board for wireless Bluetooth communication. (b) Strain sensor attached to the Adam's apple and its sensing performance during laryngeal movement. (c) Graph and (d) short‐time Fourier transform spectrogram of the MCME‐based strain sensor attached near the vocal cords in response to different voice inputs such as “One” to “Five.” Analyzing amplitude variations across frequencies enabled clear differentiation between each word. (e) Real‐time monitoring of radial artery pulse signals at the wrist under normal and post‐exercise conditions for 60 s. (f) Pulse signals recorded from 10 to 20 s and enlarged pulse waveforms showing the characteristic percussion (P‐wave), tidal (T‐wave), and dicrotic (D‐wave) peaks, demonstrating the high fidelity of the sensor.

Given their excellent sensitivity in the low‐strain range, the MCME‐based strain sensors are applied to wearable physiological monitoring (Figure 6). As indicated in Figure 6a, the fabricated wearable device is integrated with a commercial bandage and Arduino board for wireless Bluetooth communication. We confirm that the strain sensor successfully detected laryngeal movements, enabling the real‐time monitoring of swallowing (Figure 6b). The sensor is attached near the vocal cords to distinguish between different spoken words. The words from “one” to “five” are each spoken, and the corresponding relative resistance variations are recorded (Figure 6c). A short‐time Fourier transform (STFT) analysis is performed to clearly show the measured results and characterize their frequency responses. The STFT spectra reveal that vibration signals during each pronunciation contain distinct frequency components and amplitude distributions. Subsequently, STFT analysis is conducted to visualize the time–frequency evolution of the signals in a two‐dimensional spectrogram (Figure 6d). The results show that “two” and “four” exhibit relatively weak amplitudes, while “one,” “three,” and “five” show strong amplitudes. We verify the ability of the MCME‐based strain sensors to detect small deformations such as variations in the wrist pulse under normal conditions and after exercise. Continuous pulse signals were recorded for 60 s, during which 76 and 100 pulse peaks were detected under normal and post‐exercise conditions, respectively, corresponding to heart rates of 76 and 100 beats min^−^^1^ (Figure 6e). The enlarged signals from 10 to 20 s and representative pulse waveforms are shown in Figure 6f. Under normal conditions, the sensor clearly resolved the characteristic percussion (P), tidal (T), and dicrotic (D) waves of the radial pulse. After exercise, an increased pulse frequency and amplitude were observed, together with a noticeable decrease in the relative T‐wave amplitude. To further validate the reliability of wrist pulse monitoring, the pulse signal obtained from the MCME‐based strain sensor under normal conditions was compared with that measured using a commercial photoplethysmography (PPG) sensor (Figure S18). Both signals exhibited comparable waveform characteristics and pulse‐to‐pulse intervals. The corresponding heart rates measured using the MCME‐based strain sensor and PPG sensor were 76 and 74 BPM, respectively, corresponding to a relative error of approximately 2.7%. This close agreement further confirms the reliability of the MCME‐based strain sensor. These findings confirm that the fabricated MCME‐based strain sensor can not only distinguish different spoken words based on their frequency‐domain features but also detect changes in heart rate.

### 2.6 Application in Structural Health Monitoring of Aircraft Structures

**FIGURE 7:**
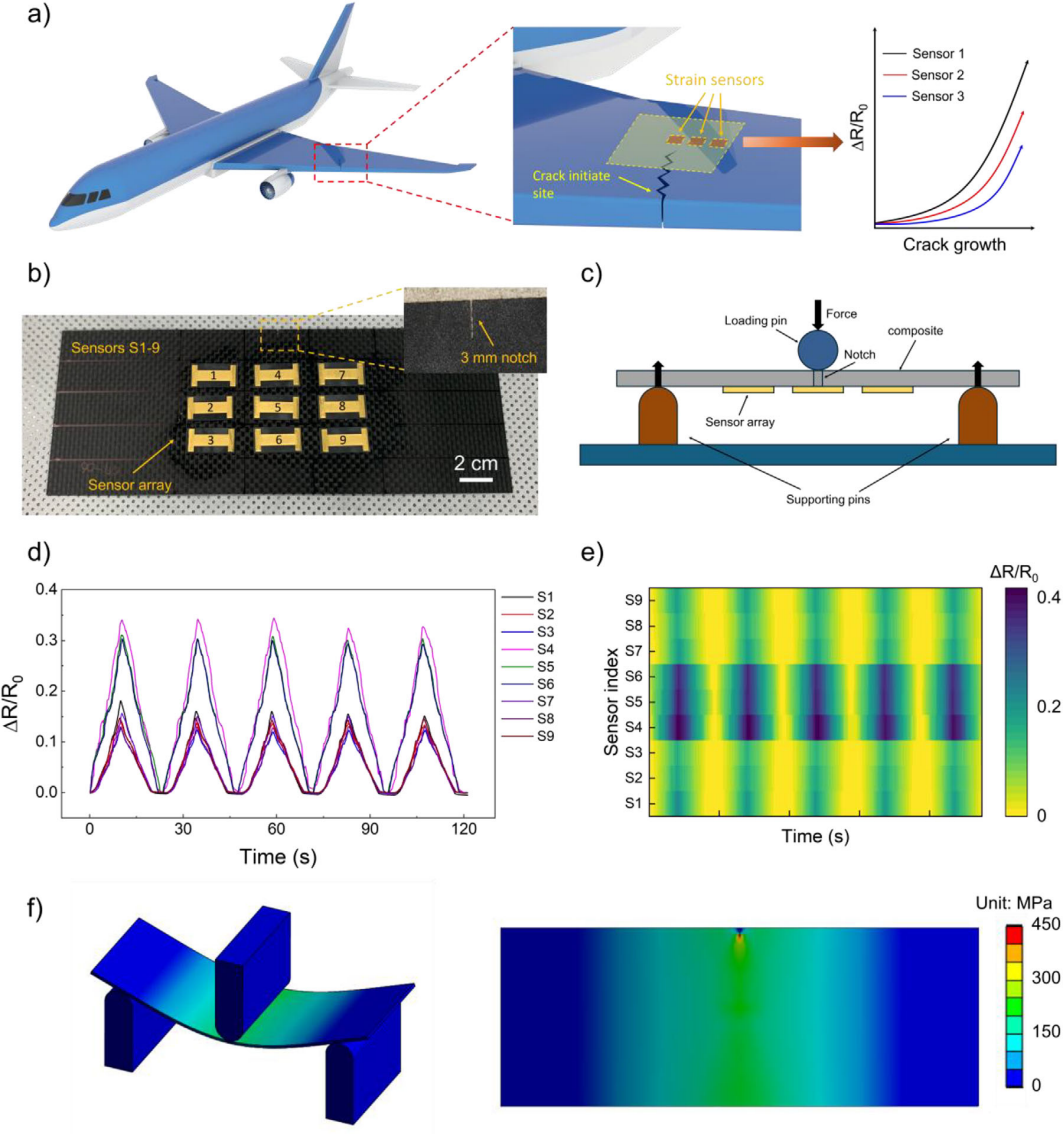
Application of the MCME‐based strain sensor for structural health monitoring of aircraft structures. (a) Conceptual illustration demonstrating the detection of crack initiation on an aircraft wing using the attached strain sensor. The graph shows the correlation between the location of the sensors and increase in the resistance signal of the sensors. (b) Photograph of the experimental setup featuring a 3 × 3 sensor array (S1–S9) integrated onto a carbon fiber reinforced plastic (CFRP) composites plate. An artificial 3 mm notch was introduced for simulating a structural defect. (c) Schematic of the three‐point bending test configuration used for applying mechanical load to the notched CFRP composite plate. (d) Real‐time relative resistance changes of the sensors under cyclic loading. (e) 2D contour map visualizing the spatial distribution of strain across the sensor array over time, which clearly indicates peak strain levels at the center where the defect is located. (f) FEA simulation of the notched CFRP composites plate under three‐point bending. The simulation confirms that mechanical stress is highly concentrated at the notch tip, which is consistent with the experimental signals obtained from the sensor array.

As shown in Figure 7, a sensor array is employed to detect structural defects in aircraft components and demonstrate the practical feasibility of the proposed MCME‐based strain sensors for real‐time structural health monitoring (SHM). CFRP composites are widely used in various mobility industries because of their high strength‐to‐weight ratio; however, they are susceptible to failure caused by cracks or delamination. Therefore, detecting the initiation and propagation of cracks at an early stage is critical to ensure safety. The proposed strain sensor exhibits high sensitivity in the low‐strain range under both static and dynamic loadings. It enables the detection of subtle deformations without requiring signal amplification systems, ensuring robust safety monitoring. Although the reliable sensing range of the present sensor is limited to 1.6% strain, transverse‐crack initiation strains reported for CFRP structures (approximately 0.41%–1.10%) fall within this range, as summarized in Table S3 [27, 28, 29]. Therefore, the proposed SHM application is primarily intended for the early detection of damage and strain redistribution, before severe local deformation occurs. Figure 7a presents a conceptual illustration of the monitoring system applied to an aircraft wing, where sensors are attached to various locations. As indicated in the graph, the sensors were designed to correlate the increase in electrical resistance signals with the location and propagation of cracks. As shown in Figure 7b, we attached a 3 × 3 sensor array (S1–S9) to the CFRP composite plate to verify this scenario experimentally. An artificial notch with a length of 3 mm was introduced at the center of the plate to concentrate the stress for comparing the signal variation relative to the sensor location. Figure 7c presents a schematic of the three‐point bending test configuration used to apply a mechanical load to the notched CFRP composite plate.

Figure 7d presents the real‐time relative resistance changes (*ΔR/R_0_*) of nine sensors under cyclic mechanical loading. The sensors exhibited distinct signal responses based on their spatial proximity to the notch. The sensors located directly above the notch tip (e.g., S4–S6) displayed the most distinct resistance changes, which reached the highest peak values. In contrast, sensors located farther away from the notch (e.g., S1–S3 and S7–S9) exhibit relatively smaller resistance changes. A two‐dimensional (2D) contour map is derived from the sensor array data (Figure 7e) to visualize the results clearly. The 2D contour map clearly indicates a “hotspot” with peak strain levels concentrated at the center of the array, precisely identifying the location of the structural defect. We performed FEA simulations on the notched CFRP model under identical loading conditions to validate the experimental findings (Figure 7f). The simulation results revealed that the mechanical stress was concentrated at the tip of the notch, creating a localized high‐stress zone. This theoretical stress distribution is in excellent agreement with the 2D contour map obtained from the sensor array. This result confirms that the MCME‐based sensor array can effectively detect subtle deformations in real time without requiring complex amplification systems because of its high sensitivity. Furthermore, these results confirm that the proposed MCME‐based strain sensors are sufficiently sensitive and reliable to function as critical components in next‐generation SHM systems for aerospace applications.

## 3 Conclusion

This study presented a bio‐inspired internal stress engineering strategy for fabricating tunable MCME. We demonstrated the formation of microscale cracks on Au by utilizing the expansion energy of thermally expandable microspheres and the thickness‐dependent brittle‐to‐ductile transition of Au thin films, which served as a sensitive sensor. This approach enabled the controlled formation of high‐density microcracks on the Au surface. The fabricated MCME‐based strain sensor exhibited exceptional sensing performance, achieving a high *GF* of ∼ 24 320 at a strain of 1.6%. FEA simulations confirmed that the enhanced sensitivity originated from the local strain distribution at the crack tips, inducing dramatic resistance changes even under subtle deformation. In addition, the sensor exhibited excellent sensing characteristics under static and dynamic cyclic loading. The MCME‐based strain sensor demonstrated versatility in real‐time wearable physiological monitoring, successfully detecting swallowing, voice, and wrist‐pulse signals. Finally, a 3 × 3 sensor array was applied to a notched CFRP plate for SHM to validate its practical applicability, successfully identifying structural deformation sites in real time without signal amplification. These results confirmed that the proposed methodology offers a new design strategy for engineering microscale cracks with high sensitivity in subtle strain ranges and holds strong potential for next‐generation strain sensors for wearable electronics, human–machine interfaces, and industrial structural health monitoring.

## 4 Experimental Section

### 4.1 Fabrication of the Microscale Cracks Induced Through Microsphere Expansion (MCME)

A silicon wafer was thoroughly cleaned using acetone, isopropyl alcohol (IPA), and deionized (DI) water under ultrasonication for 10 min each, followed by drying with nitrogen gas to remove any organic contaminants. A self‐assembled monolayer (SAM: Trichloro silane, Sigma–Aldrich, USA) was coated on the cleaned silicon wafer to prevent the elastomer from adhering to the substrate. Subsequently, the liquid elastomer (Dragon Skin, Smooth‐On, USA) was cast onto the substrate using a thin‐film applicator to form a handling layer with a thickness of 0.2 mm. Thermally expandable microspheres (Microsphere, MSH‐320, SDI Korea, Korea) were added to the well‐mixed liquid PDMS precursor (Sylgard 184, Dow Corning Corporation, USA), in which the resin and hardener were mixed in a 10:1 weight ratio. To ensure uniform dispersion and effective degassing, the mixture was processed for 90 s using a planetary mixer (KK‐V350W, Kurabo, Japan) with a revolution‐rotation ratio of 7:6. The uniformly mixed PDMS/microsphere precursor was spin coated onto the substrate at 1000 rpm for 60 s, achieving a uniform thickness of approximately 100 µm. Thermal curing was then performed at 70°C for 2 h. A thin Au film was subsequently deposited via sputtering through a high‐precision shadow mask to define the sensor pattern. Finally, the device was thermally treated at 170°C for 5 min, a temperature corresponding to the maximum expansion of the microspheres. This rapid volumetric expansion induced significant internal stress within the metal film, leading to the spontaneous generation of microscale surface cracks.

### 4.2 Fabrication of the MCME‐Based Strain Sensor

For electrode fabrication, copper foil tape (1181, 3M, USA) was used as the conductive electrode, and silver epoxy (CW‐2400, ITW Chemtronics, USA) was applied to attach the electrodes. The assembled devices were then cured in an oven at 80°C for 30 min to ensure stable bonding. Subsequently, electrical sensing wires were connected to the copper electrodes for subsequent sensing measurements.

### 4.3 Characterization of the MCME

The surface morphology of the MCME was characterized using scanning electron microscopy (SNE‐ALPHA, Scientific Equipment Company, Korea) and optical microscopy (BX53M, Olympus Corporation, Japan). High‐resolution sample images were captured using a camera equipped with 3D‐DIC stereo microscopy (NFEC‐2026‐01‐311924). The tensile tests were conducted using a universal testing machine (UTM) (AGS‐X (10 kN), Shimadzu Corporation, Japan) equipped with a 10 kN load cell. All tests were performed at a crosshead rate of 10 mm min^−1^ except for the frequency response test. To evaluate the sensor performance of MCME‐based strain sensors, resistance variation (Δ*R*/*R*_0_) was measured using an LCR meter (IM3536, Hioki, Japan) with an AC bias of 1 V. All sensor evaluations were measured by connecting the LCR meter to a computer in real time. All measurements were carried out at room temperature (24°C) under ambient conditions.

### 4.4 Application of the MCME‐Based Strain Sensor for Physiological and Structural Health Monitoring

The physiological signals were obtained as follows. An MCME‐based strain sensor with copper tape attached to both ends was prepared, and copper wires were connected to each electrode to form the sensor assembly. For the measurement of physiological signals such as swallowing, voice, and wrist pulse, the sensor assembly was attached to the neck or wrist using a soft elastic bandage. A Bluetooth module (HC‐06), a battery, and an Arduino Uno were used to measure the physiological signals wirelessly. For validation of wrist pulse monitoring, a commercial PPG sensor (MAX30102) was additionally used as a reference measurement system. The PPG signal was inverted to match the waveform direction of the MCME‐based strain sensor, thereby facilitating intuitive comparison between the two signals. Both signals were normalized to a range of 0–1 for direct comparison in Figure S18, whereas the non‐normalized MCME‐based strain sensor signal is presented in Figure 6f. All experiments involving human subjects were conducted with approval from the Institutional Review Board (IRB) of the Pukyong National University (PKNU) (IRB no. 2025‐08‐019). For the structural health monitoring of aircraft structures, nine MCME‐based strain sensors were attached to a commercial carbon fiber reinforced plastic (CFRP) composite plate. An artificial 3 mm notch was introduced to simulate a structural defect.

### 4.5 Finite Element Modeling and Analysis

FEA was performed to investigate the mechanical behavior associated with microsphere‐induced localized internal stress, tensile deformation behavior of a cracked Au thin film, and three‐point bending of a notched CFRP specimen. The microsphere‐based models were uniformly scaled by 1000× to facilitate meshing of the nanoscale Au thin film. The detailed material parameters and mesh conditions are provided in Table S4.

First, a 2D microsphere‐expansion model consisting of a PDMS substrate, an embedded microsphere, and an Au thin film was constructed to evaluate the equivalent stress distribution in the Au thin film. The microsphere was positioned 15 and 35 µm below the Au thin film. PDMS was modeled using a five‐parameter Mooney–Rivlin model based on literature‐reported parameters [30]. The Au thin film was modeled as a separate layer, and the Au–PDMS interface was defined as bonded. The bottom surface of the PDMS substrate was fixed. Because the exact mechanical properties of the microsphere (MSH‐320) were unavailable, the microsphere expansion was prescribed as an approximately threefold increase in diameter relative to the initial state.

Second, a 2D tensile model was constructed to evaluate the strain distribution around a crack. The cracked geometry was modeled based on the crack morphology observed in SEM images. The crack was positioned at the apex of the Au thin film directly above the expanded microsphere. Total tensile strains of 0.8% and 1.6% were applied by imposing symmetric displacements on both lateral sides of the model, while the bottom surface was constrained in the vertical direction.

Third, a 3D three‐point bending model was constructed to evaluate the localized stress distribution around a notch in a CFRP specimen. The CFRP specimen was modeled using orthotropic elastic properties. The CFRP–jig interfaces were defined as frictional contact with a friction coefficient of 0.2. The two lower supports were fixed, and a downward displacement of 20 mm was applied to the central loading jig.

## Author Contributions

**Jae Yeong Jang**: conceptualization, methodology, software, data curation, investigation, validation, formal analysis, visualization, writing – original draft. **Hanchul Cho**: data curation, validation, investigation. **Young Jung**: conceptualization, writing – review and editing, resources, project administration, funding acquisition, supervision.

## Conflicts of Interest

The authors declare no conflicts of interest.

## Supporting information




**Supporting File**: advs77961‐sup‐0001‐SuppMat.docx.

## Data Availability

The data that support the findings of this study are available from the corresponding author upon reasonable request.
