# Supplementary material for: Bio‐Inspired Internal Stress Engineering for Microscale Surface Cracking in Sensitive Strain Sensors
Source: Adv Sci (Weinh). 2026 Sep 27:e77961. Online ahead of print. doi: 10.1002/advs.77961 (PMC13616271; doi:10.1002/advs.77961)
Supplement: Supplementary file 1 [file ADVS-9999-e77961-s001.docx]

**Supporting Information**

Bio-inspired Internal Stress Engineering for Microscale Surface Cracking in Sensitive Strain Sensors

Jae Yeong Jang, Hanchul Cho, Young Jung

**Table S1.** Electrical resistance of Au and Pt films with different thicknesses before and after microsphere expansion at a microsphere concentration of 3 wt%.

| **Materials** | **Thickness** | **Resistance** | |
| --- | --- | --- | --- |
|  |  | **Before expansion** | **After expansion** |
| Au | 20 nm | 111.7 ± 31.83 Ω | Not measurable |
|  | 40 nm | 1.98 ± 0.19 Ω | 20.5 ± 6.93 Ω |
|  | 60 nm | 2.08 ± 0.05 Ω | 2.93 ± 0.06 Ω |
| Pt | 20 nm | 174.8 ± 16.2 Ω | 181.21 ± 10.36 Ω |
|  | 40 nm | 37.30 ± 1.80 Ω | 38.9 ± 5.54 Ω |
|  | 60 nm | 7.36 ± 0.25 Ω | 8.80 ± 0.19 Ω |

**Table S2.** Comparison of the present MCME-based strain sensor with recently reported crack-based strain sensors.

| **Structure** | **Sensing Materials** | **GF/strain ranges (%)** | | | **Response time (ms)** | **Reliability** | **Ref.** |
| --- | --- | --- | --- | --- | --- | --- | --- |
|  |  | **Low** | **Medium** | **High** |  |  |  |
| Wrinkled/ crack-bridging layer | CNT/GO | 287.6 (0-100) | - | - | 489 | 10000 cycles at 80% strain | [19] |
| Auxetic  meta-crack | Mo | 1450 (0-0.01) | 7720 (0.01-0.29) | - | - | 50 cycles up to 0.29% | [8] |
| Hierarchical cracks | Au/graphene | 153 (0-20) | - | - | 42.5 | 1500 cycles | [20] |
| Cracked Au/ AgNW micromesh | Au/AgNWs | 2658.9 (0-16) | 11026 (16-20) | - | 180 | 1000 cycles at 10% strain | [21] |
| Aligned crack/ 3D vertical graphene network | Graphene | 5200 (0-2) | - | - | 200 | 10000 cycles at 2% strain | [22] |
| Crack-bridged porous network | BP/LEG | 81.2 (0-7.5) | 302.7 (7.5-19.2) | 2765 (>19.2) | 83.6 | 18400 cycles at 10% strain | [23] |
| 3D crack | Graphite | 1084.16 (0-100) | - | - | 489 | 1000 cycles at 3% strain | [24] |
| Crack-based serpentine structure | Au | 321.63 (0-200) | - | - | 103 | 1000 cycles at 50% strain | [25] |
| Archimedean spiral crack/ micropore | TPU/CNTs/  GNS | 97.35 (0-0.7) | 218.13 (0.7-2) | - | 350 | 5000 cycles at 0.3% strain | [26] |
| MCME | Au/MS | 1275 (0–0.8) | 2617 (0.8–1.2) | 24320 (1.2–1.6) | 305 | 5000 cycles at 0.7% strain | This work |

**Table S3.** Comparison of reported crack-initiation strains in CFRP structures

| **Study** | **CFRP configuration / test condition** | **Crack-initiation strain** | **Ref.** |
| --- | --- | --- | --- |
| Okabe et al., 2015 | Cross-ply CFRP under tensile loading | ≈ 0.65–0.70% | [27] |
| Hosoi et al., 2015 | CFRP laminate under tensile loading | ≈ 0.6% | [28] |
| Szpoganicz et al., 2025 | CFRP laminates  (different temperature conditions) | 0.41%, 1.03%, 1.10% | [29] |

**Table S4**. Material properties and mesh sizes used in the finite element simulations. The microsphere-based models were uniformly scaled by a factor of 1000.

| **Material** | **Young’s modulus (GPa)** | **Poisson’s ratio** | **Hyperelastic parameters** | **Mesh size (mm)** | **Remarks** |
| --- | --- | --- | --- | --- | --- |
| PDMS | - | - | C_10_ = 0.34 MPa, C_01_ = -0.27 MPa,  C_20_ = 0.32 MPa, C_11_ = - 0.14 MPa,  C_02_ = 0.23 MPa, D_1_ = 0 MPa^-1^ | 1 | [30] |
| Au film | 75.76 | 0.42 | - | 0.05 | Pure gold |
| CFRP | E_x_ = 290,  E_y_ = 23,  E_z_ = 23 | ν_xy_ = 0.20,  ν_yz_ = 0.40,  ν_zx_ = 0.20 | - | 3 | - |

**Figure S1.** Thermal expansion mechanism of thermally expandable microspheres. Upon heating, the thermoplastic shell softens (state 2), followed by vaporization of the encapsulated hydrocarbon core (state 3), which increases the internal pressure and induces substantial volumetric expansion of the microsphere (state 4). Upon cooling, the shell resolidifies and retains the expanded morphology.

**Figure S2.** Long-term retention of the expanded microsphere morphology. Scanning Electron Microscope (SEM) images of active layers containing i) 1, ii) 3, and iii) 5 wt% microspheres immediately after thermal expansion (top) and after storage at room temperature for 10 days (bottom). The expanded microsphere-induced surface 3D structures remained clearly visible after storage, demonstrating the retention of the expanded morphology over time.

**Figure S3.** Large-area SEM images and corresponding crack-density analyses under different conditions. (a–d) Effect of Au-film thickness at 3 wt% microsphere concentration, (e–h) effect of microsphere concentration for a 40-nm-thick Au film, and (i–l) effect of Pt-film thickness at 3 wt% microsphere concentration.

**Figure S4.** FEA analysis of the effect of microsphere depth on the localized stress in the Au thin film. Microspheres located closer to the Au film induce higher local stress, while increasing the microsphere depth reduces the stress transferred to the film.

**Figure S5**. SEM images of microscale cracks. Microscale cracks are generated and propagated on Au thin films with microsphere expansion.

**Figure S6.** Surface distribution of expanded microspheres in active layers with microsphere concentrations of 1, 3, and 5 wt%. a) Representative SEM images at i) 1, ii) 3, and iii) 5 wt%. b) Corresponding thresholded images used to quantify the area fraction of microsphere-induced surface 3D structures. The red regions in the thresholded images indicate the surface area used for the analysis of area fraction.

**Figure S7.** Size distributions of expanded microspheres in active layers with microsphere concentrations of 1, 3, and 5 wt%. Gaussian distribution of the diameter of the expanded microspheres in the active layers was obtained from SEM images of the active layers containing i) 1 wt%, ii) 3 wt%, and iii) 5 wt% of microspheres. The three conditions exhibit comparable peak positions and distribution widths, indicating similar characteristic size distributions of the expanded microspheres over the investigated concentration range.

**Figure S8.** Microscale cracks generated in the Au thin film by mechanical pre-stretching. The cracks exhibited irregular morphology with nonuniform spacing and propagation directions, illustrating the limited control over crack formation achieved by mechanical pre-stretching.

**Figure S9**. Dynamic response of the MCME-based sensor under various applied strains at 2.0%. At 2.0% strain, the gaps in the microscale cracks increased, resulting in unreliable and inconsistent resistance variation.

**Figure S10**. Comparison of the dynamic response of the FM and MCME-based sensors at 0.8% strain: a) The FM-based sensor shows a low gauge factor of 24. b) The MCME-based sensor exhibits a significant resistance variation as 306, which is 12 times higher than that of the FM-based sensor.

**Figure S11**. Sensor-to-sensor reproducibility of ten independently fabricated MCME-based sensors. They exhibited similar responses with negligible deviations.

**Figure S12.** Size distributions of expanded microspheres in active layers: a) between multiple regions within a single 3 wt% sample; b) between multiple 3 wt% samples. The three regions within a single sample and three independently fabricated samples exhibited approximately similar microsphere size distributions.

**Figure S13.** Sensing performance of MCME-based strain sensors with different microsphere concentrations. Relative resistance changes (Δ*R*/*R*_0_) of sensors containing 1, 3, and 5 wt% microspheres as a function of applied strain.

**Figure S14.** Dynamic response of the Pt-based strain sensor under various applied strains at a) 0.4% and b) 0.8% strain. The progressive increase in signal amplitude during repeated deformation indicates an unstable electrical response of the Pt film under cyclic loading.

**Figure S15.** Response time of the MCME-based strain sensor for an applied strain of 0.8%. The 10% to 90% rising time (*t_r_*) and the 90% to 10% falling time (*t_d_*) are 305 and 416 ms, respectively.

**Figure S16.** The linear calibration curve of the strain sensor responses for the calculation of limit of detection (LOD). The LOD was calculated from the standard deviation of the normalized baseline signal (*σ*) and the sensitivity in the low-strain region using $LOD={3\sigma}/S$. We confirmed that the theoretical LOD of our strain sensor is as low as 0.070% strain.

**Figure S17.** Long-term cyclic stability of the MCME-based strain sensor over 5,000 loading–unloading cycles at an applied strain of 0.7%. The sensor maintained a stable cyclic response without abrupt signal loss or electrical failure.

**Figure S18.** Comparison of radial pulse signals measured using the photoplethysmography (PPG) sensor and the MCME-based strain sensor under normal conditions a) over 60 s and b) during the initial 6 s.
